# Slow Flow Phenomenon Impairs the Prognosis of Coronary Artery Ectasia as Well as Coronary Atherosclerosis

**DOI:** 10.21470/1678-9741-2020-0618

**Published:** 2021

**Authors:** Mehmet Kaplan, Özge Özcan Abacıoğlu, Fethi Yavuz, Gizem Ilgın Kaplan, Mustafa Topuz

**Affiliations:** 1 Department of Cardiology, Gaziantep University, Gaziantep, Turkey.; 2 Department of Cardiology, Adana City Training & Research Hospital, Adana, Turkey.; 3 Department of Internal Medicine, Ersin Arslan Training & Research Hospital, Gaziantep, Turkey.

**Keywords:** Coronary Vessels, Constriction, Pathologic, Coronary Stenosis, Coronary Aneurysm, Angiography, Hospitalization

## Abstract

**Introduction:**

Coronary artery ectasia (CAE) is one of the uncommon cardiovascular disorders and its prognosis is still debated.

**Objective:**

We aimed to review long-term follow-up data in patients with CAE and to evaluate the prognosis of CAE patients with coronary slow flow phenomenon (CSFP).

**Methods:**

This study had a prospective design and 143 patients with CAE were included. The angiographic and demographic characteristics were reviewed in detail. The patients were categorized, based on concomitant coronary artery stenosis detected by angiography, as CCAE group (n=87, ≥30% luminal stenosis) and ICAE group (n=56, <30% luminal stenosis) and also categorized by the coronary flow as CSFP group (n=51) and normal flow coronary ectasia - NCEA group (n=92). All patients were re-evaluated at 6-month intervals. Follow-up data, cardiovascular (CV) mortality, hospitalization and major adverse cardiac events (MACE) were collected. The level of statistical significance was set at 5%.

**Results:**

Patients were followed up for an average of 56.9±7.4 months. During the follow-up, statistically significant differences were found in hospitalization, CV mortality and MACE between the CCAE and ICAE groups (*P*=0.038, *P*=0.003, *P*=0.001, respectively). The CSFP and NCEA groups were also compared. There was a statistical difference with respect to hospitalization between the CFSP and NCEA groups (*P*=0.001), but no difference was observed in terms of MACE and CV mortality (*P*=0.793 and *P*=0.279).

**Conclusion:**

CSFP accompanying CAE may be a predictor of hospitalization. Significant atherosclerotic plaques coexisting with CAE may be predictive for MACE.

**Table:** 

Abbreviations, acronyms & symbols				
**ACS** **CABG** **CAD** **CAE** **CCAE** **CRP** **CSFP** **CV** **Cx** **EF** **HIV** **HT** **ICAE** **IGF-I**	**= Acute coronary syndrome** **= Coronary artery bypass grafting surgery** **= Coronary artery disease** **= Coronary artery ectasia** **= Coronary artery ectasia with ≥30% luminal stenosis** **= C-reactive protein** **= Coronary slow flow phenomenon** **= Cardiovascular** **= Circumflex artery** **= Ejection fraction** **= Human immunodeficiency virus** **= Hypertension** **= Isolated coronary artery ectasia** **= Insulin-like growth factor I**		**LAD** **LMCA** **MACE** **MHR** **NCEA** **NLR** **PCI** **RCA** **SPSS** **TNF-α** **QCA** **TIMI** **TFC** **UA**	**= Left anterior descending artery** ** = Left main coronary artery** **= Major adverse cardiac events** **= Monocyte to high-density lipoprotein ratio** **= Normal flow coronary ectasia** **= Neutrophil-to-lymphocyte ratio** **= Percutaneous coronary intervention** **= Right coronary artery** **= Statistical Package for the Social Sciences** **= Tumor necrosis factor alpha** **= Quantitative coronary analysis** **= Thrombolysis in myocardial infarction** **= TIMI frame count** **= Uric acid**

## INTRODUCTION

Coronary artery ectasia (CAE) is described as the dilation of a segment of a coronary artery at least 1.5 times the adjacent segment ^[1]^. CAE and aneurysms are encountered in 1.5-5% of coronary angiographies, but are less frequent in non atherosclerotic cases ^[2]^. The exact pathophysiological mechanisms of CAE have not been clearly understood. A variety of etiologies, including congenital defects, inﬂammation, endothelial dysfunction, vasculitis and atherosclerosis are associated with the development of CAE, but atherosclerosis is the main cause in nearly half of all CAE cases ^[3,4]^. In the clinical setting, CAE is associated with adverse cardiovascular outcomes, such as coronary spasm, thrombosis, distal embolization, dissection, and myocardial ischemia ^[5]^.

Isolated coronary artery ectasia (ICAE) is defined as the absence of coronary artery stenosis and accounts for roughly 0.1-0.79% of all CAE cases ^[6]^. In addition to atherosclerosis, slow flow may also accompany ICAE. Higher rates of morbidity and mortality were found in CAE patients with atherosclerosis than in ICAE patients in previous studies ^[7]^. There are limited data in the literature regarding CAE and especially its prognosis due to its low incidence.

This study investigated the long-term clinical outcomes of a sample of CAE patients from southeastern Anatolia. In addition, we aimed to evaluate the prognosis of CAE patients with coronary slow flow phenomenon (CSFP).

## METHODS

This study had a prospective design and included complete follow-up until June 2019 for patients with CAE who underwent coronary angiography between January 2008 and January 2013 at the Adana City Research and Training Hospital. Study patients were selected among patients who presented to our clinic with stable angina pectoris and were subsequently referred to coronary angiography.

Patients were categorized based on concomitant coronary artery stenosis detected by angiography as CCAE group (n=87, ≥30% luminal stenosis) and ICAE group (n=56, <30% luminal stenosis), and also categorized by the coronary flow as CSFP group (n=51) and normal flow coronary ectasia - NCEA (n=92). The clinical, angiographic and demographic characteristics of the patients were reviewed in detail. These groups were compared in terms of major adverse cardiac events (MACE), hospitalization and cardiovascular (CV) mortality.

The exclusion criteria were: severe or moderate valvular heart disease, percutaneous coronary intervention (PCI) on ectatic vessel, heart failure (left ventricular ejection fraction <40%), inﬂammatory diseases (acute or chronic), and hepatic and renal disorders. In addition, patients for whom detailed information (no data on 6-month follow-up) could not be retrieved from their medical records were excluded from the study.

CV risk factors for coronary artery disease (CAD) were identified from patients’ hospital records and physical examination at the time of presentation, including hypertension (HT), diabetes mellitus, current or past smoking, family history of CAD and dyslipidemia. We used standard definitions for risk factors, as described in current guidelines. The study was approved by the Institutional Review Board of the Adana City Research and Training Hospital.

### Coronary Angiography

The images were evaluated by two clinical specialists who were blinded to the clinical findings of the patients. Coronary angiography was performed with Philips Allura Xper FD10 device (Philips Medical Systems). Femoral artery cannulation and Judkins method were used for coronary angiography. The radiocontrast agents were iodixanol or iohexol. Two orthogonal projections were recorded and analysed by two interventional cardiologists using the software of Philips Allura Xper FD10 device. CAE was defined for coronary angiography findings of segmental or diffuse coronary artery dilation to >1.5-fold the diameter of adjacent segments of the same artery without stenosis. Ectatic vessel diameter and ratio of the ectatic segment to the normal segment diameter were determined using the quantitative coronary analysis (QCA) system. Using this method, CAE patients were divided into two groups, including the ICAE group (<30% luminal stenosis) and the CCAE group (≥30% luminal stenosis). In addition, the degree of CAE was categorized according to the Markis classification as follows: type 1, diffuse ectasia in two or three vessels; type 2, diffuse ectasia in one vessel and localized disease in another vessel; type 3, diffuse ectasia in a single vessel; and type 4, segmental localized ectasia ^[8]^.

CSFP is an angiographic entity characterized by delayed progression of the injected contrast medium through the coronary tree. Two interventional cardiologists calculated thrombolysis in myocardial infarction (TIMI) frame counts (TFCs) for each coronary vessel. Images were acquired at 15 frames/s, and all frames were multiplied by 2. The left anterior descending (LAD) artery frame counts were divided by 1.7 due to the longer length. Similar to previous established methods, a frame count above 27 for all vessels was considered a slow coronary flow ^[9]^. According to these criteria, 51 patients were included in the CSFP group.

### Follow-up

The eligible patients were re-evaluated at 6-month intervals. Patients whose detailed data could not be retrieved and those who did not return for follow-up were excluded from the study. Follow-up examinations were conducted at the hospital during routine check-ups. For a small number of patients (8%), follow-up data were obtained from the hospital or health center registry, from clinical notes, or from telephone surveys conducted by two cardiologists. Although the follow-up process started with 968 evaluable patients, complete follow-up was achieved only for 143 patients.

Complete follow-up data, including information on MACE (recurrent interventions, acute coronary syndromes, PCI and coronary artery bypass grafting [CABG] surgery), hospitalization and CV mortality were collected. Hospitalization was defined as hospital stay (≥2 days) for stable angina and recurrent intervention was defined as need for intervention (e.g., PCI or CABG) in an ectatic vessel due to angina pectoris. At least one culprit lesion was found in the ectatic vessels in acute coronary syndrome (ACS) and PCI cases. Cardiovascular mortality was defined as a failed intervention on an ectatic vessel and subsequent heart failure or cardiac arrest associated with malignant arrhythmias.

### Statistical Analysis

The SPSS for Windows software (version 22.0, IBM Corp., Armonk, NY, USA) was used for all statistical analyses. A Kolmogorov-Smirnov test was used to check the normality of the distributions of continuous variables. The independent samples t-test was used to assess normally distributed variables and the Mann-Whitney U test was used for non-normally distributed variables. Mean±standard deviation or median (interquartile range) were calculated for continuous variables depending on whether they showed a normal distribution. Categorical variables were presented as percentages. Follow-up data (without follow-up variable) from CCAE-ICAE and NCEA-CSFP groups were compared using the chi-square test. The common variable (hospitalization) considered significant by these tests was included in the binomial logistic regression analysis as a dependent variable, and the effect of significant ectatic coronary lesions and the coronary slow phenomenon on this dependent variable were investigated. A two-sided P<0.05 was considered statistically significant in all statistical analyses.

## RESULTS

CAE was observed in one vessel in 46.1% (n=66), in two vessels in 47.5% (n=68) and in three vessels in 6.4% (n=9) of the patients. Of all arteries, the right coronary artery (RCA) was the vessel most affected by ectasia (in 64.3% of patients, irrespective of the number of affected coronary vessels) and type 1 ectasia was the most common (30%) according to the criteria of Markis et al. ^[8]^ (Table 1). No significant difference in age or sex was observed when the demographics of the groups were evaluated. In addition, the groups were similar in terms of diabetes mellitus, HT, smoking, dyslipidemia and family history for CAD, with no statistically significant difference (*P*>0.05). There was no significant difference in biochemical variables between the study groups based on a comparison of the laboratory data (*P*>0.05) (Table 2).

**Table 1:** Ectatic lesions, coronary vessels and Markis classification.

Vessel	CCAE (n=87)	ICAE (n=56)	Total (n=143)
LAD (n, %)	8 (9.2%)	8 (14.3%)	16 (11.2%)
Cx (n, %)	9 (10.3%)	6 (10.7%)	15 (10.5%)
RCA (n, %)	21 (24.1%)	13 (23.2%)	34 (23.8%)
LAD-Cx (n, %)	7 (8%)	12 (21.4%)	19 (13.3%)
LAD-RCA (n, %)	10 (11.5%)	4 (7.1%)	14 (9.8%)
Cx-RCA (n, %)	23 (26.4%)	12 (21.4%)	35 (24.5%)
LAD-Cx-RCA (n, %)	8 (9.2%)	1 (1.8%)	9 (6.5%)
LMCA (n, %)	1 (1.1%)	0 (0%)	1 (0.07%)
**Markis classification**			
Type 1 (n, %)	26 (29.9%)	17 (30.4%)	43 (30.1%)
Type 2 (n, %)	22 (25.3%)	12 (21.4%)	34 (23.8%)
Type 3 (n, %)	14 (16.1%)	13 (23.2%)	27 (18.9%)
Type 4 (n, %)	25 (28.7%)	14 (25.0%)	39 (27.3%)

CAD=coronary artery disease; Cx=circumflex artery; CCAE= ≥30% luminal stenosis - coronary ectasia; ICAE=isolated coronary artery ectasia; LAD=left anterior descending artery; LMCA=left main coronary artery; RCA=right coronary artery

**Table 2:** Baseline demographic characteristics and biochemical variables of the CAE groups.

Demographic characteristics	CCAE (n=87)	ICAE (n=56)	*P* value
Age (years, mean)	52.3 ±9.2	54.89 ±10.7	0.281
Gender (female, %)	44 (50.5%)	28 (50%)	0.947
HT (n, %)	37 (42%)	27 (48%)	0.505
Diabetes mellitus (n, %)	29 (33%)	14 (25%)	0.289
Smoking (n, %)	43 (49%)	21 (37%)	0.162
Dyslipidemia (n, %)	48 (55%)	30 (53%)	0.865
Family history for CAD (n, %)	36 (41%)	19 (34%)	0.371
**Biochemical variables**			
Hemoglobin (g/dL)	13.1±1.8	12.7±1.6	0.196
Leukocytes (×10^3^/µL)	7.59±1.7	7.45±1.7	0.635
Monocytes (×10^3^/µL)	0.56 (0.40)	0.6 (0.31)	0.775
Platelets (×10^3^/µL)	286±70	295±83	0.488
Fasting plasma glucose (mg/dL)	95 (94)	96 (68)	0.309
Creatinine (mg/dL)	0.69 (0.21)	0.70 (0.28)	0.733
LDL-C (mg/dL)	135 (41)	134 (37)	0.823
HDL-C (mg/dL)	38 (18)	41(12)	0.843
AST (IU/L)	18 (6)	19 (7)	0.358
ALT (IU/L)	19 (8)	17 (6)	0.488

ALT=alanine aminotransferase; AST=aspartate aminotransferase; CAD=coronary artery disease; CCAE=≥30% luminal stenosis - coronary ectasia; HDL-C=high-density lipoprotein cholesterol; HT=hypertension; ICAE=isolated coronary artery ectasia; LDL-C=low-density lipoprotein cholesterol

The patients were followed up for an average period of 56.9±7.4 months. Compared with respect to hospitalization, 12 patients in the ICAE group and 33 patients in the CCAE group were hospitalized secondary to cardiac causes, and the difference was statistically significant (*P*=0.044). In addition, cardiovascular mortality, PCI and acute coronary syndromes were statistically significantly more common in the CCAE group (*P*<0.05). In contrast, 16 patients in the CCAE group and 4 patients in the ICAE group underwent CABG surgery, with no statistically significant difference (*P*=0.083) (Table 3).

**Table 3:** Follow-up data of the groups.

	CCAE (n=87)	ICAE (n=56)	*P* value
Hospitalization (n, %)	33 (38%)	12 (21%)	0.038*
MACE (n, %)	37 (43%)	7 (13%)	0.001*
Cardiovascular mortality (n, %)	12 (14%)	0 (0%)	0.003*
PCI (n, %)	28 (32%)	6 (11%)	0.003*
Coronary bypass surgery (n, %)	16 (18%)	4 (7%)	0.058
ACS (n, %)	9 (10%)	0 (0%)	0.013*
Follow-up (years, mean)	57.1 ±7.7	56.8 ±7.2	0.793

ACS=acute coronary syndrome; CAD=coronary artery disease; CCAE= ≥30% luminal stenosis - coronary ectasia; ICAE=isolated coronary artery ectasia; MACE=major adverse cardiac events; PCI=percutaneous coronary intervention

* A *P* <0.05 is considered significant.

During the follow-up, 12 (8.4%) patients died, all of them in the CCAE group, and the deaths occurred as a result of cardiac causes (acute coronary syndromes followed by ventricular fibrillation in 8 patients, decompensated heart failure in 1 patient, and malignant arrhythmia in 3 patients). This led to a statistically significant difference in mortality between the groups (*P*=0.003).

Slow flow was observed in one vessel in 60.7% (n=33), two vessels in 33.3% (n=17), and three vessels in 5.8% (n=3) of the patients followed. Of all arteries, RCA was the most affected by coronary slow flow (68.6%), followed by LAD and circumflex artery (Cx) in decreasing order. No statistical difference was observed between the CFSP and NCEA groups in terms of demographic characteristics, including diabetes, HT, smoking, dyslipidemia and family history of CAD (*P*>0.05). When these groups were evaluated in terms of laboratory data, there was no significant difference between the groups. Relevant data are shown in Table 4.

**Table 4:** Baseline demographic characteristics and biochemical variables of the CAE groups.

Demographic characteristics	NCEA (n=92)	CSFP (n=51)	*P* value
Age (years, mean)	54 ±9.2	52±10	0.138
Gender (female, %)	50 (54%)	22 (43%)	0.199
Hypertension (n, %)	37 (40%)	27 (53%)	0.143
Diabetes mellitus (n, %)	28 (30%)	15(29%)	0.898
Smoking (n, %)	45 (49%)	19(37%)	0.179
Dyslipidemia (n, %)	52 (56%)	26 (51%)	0.524
Family history for CAD (n, %)	31 (33 %)	24 (47%)	0.116
**Biochemical variables**			
Hemoglobin (g/dL)	13±1.8	13±1.7	0.653
Leukocytes (×10^3^/µL)	7.7±1.7	7.3±1.7	0.232
Monocytes (×10^3^/µL)	0.58 (0.39)	0.5 (0.34)	0.531
Platelets (×10^3^/µL)	283 (90)	292 (76)	0.629
Fasting plasma glucose (mg/dL)	96 (83)	96 (88)	0.992
Creatinine (mg/dL)	0.7 (0.2)	0.67 (0.3)	0.137
LDL-C (mg/dL)	134 (36)	146 (42)	0.613
HDL-C (mg/dL)	38 (16)	42 (19)	0.504
AST (IU/L)	18 (6)	19 (7)	0.147
ALT (IU/L)	17 (7)	19 (5)	0.227

ALT=alanine aminotransferase; AST=aspartate aminotransferase; CAD=coronary artery disease; CSFP=coronary slow flow phenomenon; HDL-C=high-density lipoprotein cholesterol; LDL-C=low-density lipoprotein cholesterol; MACE=major adverse cardiac events; NCEA=normal flow coronary ectasia

Of the 12 patients who died, 6 were in the NCEA group, while the other 6 patients were in the CSFP group. The difference between the groups in terms of cardiovascular mortality was not statistically significant (*P*=0.376). Fifteen patients in the NCEA group and 30 patients in the CSFP group were hospitalized due to cardiac causes (particularly stable angina pectoris), which led to a statistically significant difference in hospitalization between the groups (*P*=0.001). The rates of PCI, CABG and ACS did not differ significantly between the groups (*P*>0.05) (Table 5).

**Table 5:** Follow-up data of the groups.

	NCEA (n=92)	CSFP (n=51)	
Hospitalization (n, %)	15 (16%)	30 (59%)	0.001*
MACE (n, %)	29 (32%)	15 (29%)	0.793
Cardiovascular mortality (n, %)	6 (7%)	6 (12%)	0.279
PCI (n, %)	22 (24%)	12 (24%)	0.959
Coronary bypass surgery (n, %)	12 (13%)	8 (16%)	0.663
ACS (n, %)	6 (7%)	3 (6%)	0.880
Follow-up (years, mean)	57 ±7.6	58 ±7.4	0.290

ACS=acute coronary syndrome; CSFP=coronary slow flow phenomenon; MACE=major adverse cardiac events; NCEA=normal flow coronary ectasia; PCI=percutaneous coronary intervention

* A *P* <0.05 is considered significant.

Among the 51 CFSP patients, 31 had significant concomitant coronary artery stenosis (>30%) and MACE and hospitalization rates were statistically higher in this group in comparison to the CSFP group without significant coronary artery stenosis (*P*=0.03 and *P*=0.02, respectively). However, no difference was observed between these groups in CV mortality (*P*=0.152).

In light of the aforementioned data, the impacts of significant ectatic coronary lesion and CSFP on the odds ratio of hospitalization were examined with a binomial logistic regression analysis. According to the results of this regression analysis, concomitant CSFP increased the relative risk of hospitalization by more than 2.8 times in CAE patients in comparison to atherosclerosis. The results are shown in Table 6.

**Table 6:** Relationship of ectatic coronary lesion and coronary slow flow with hospitalization.

Term	estimates with standard errors	OR	*P* value
Constant (β_0_)	-2.407±0.454	-	<0.001*
Significant ectatic coronary lesion (β_1_) (≥30% luminal stenosis)	1.097±0.451	2.995	0.015*
CSFP (β_2_)	2.141±0.454	8.504	<0.001*

CSFP=coronary slow flow phenomenon

* A *P* <0.05 is considered significant.

In the case of CAE, no significant difference was observed between groups in terms of age, gender and biochemical data when patients who underwent repeated interventions (44 patients) were compared with those who did not undergo repeated interventions (99 patients) (*P*>0.05). In addition, the groups were similar in terms of major CAD risk factors, with no statistical difference (*P*>0.05). These patient groups did not show a statistically significant difference with respect to the slow flow (*P*=0.852). However, the presence of significant ectatic lesions was statistically significant in patients with a history of repeated interventions (*P*=0.001). Ejection fraction was lower in the group with repeated interventions, and the difference was also statistically significant (*P*=0.006). CV mortality rate was higher and statistically significant in the group undergoing repeated interventions (*P*=0.001).

## DISCUSSION

In the current study, we first identified CAE patients and then we evaluated the coexistence of CSFP and/or coronary artery disease with CAE. Finally, we investigated the prognosis of CAE patients over a period of approximately 60 months on average. In this respect, this is the first study in literature that clearly demonstrated the effect of the CSFP on the prognosis of CAE patients.

The frequency of CAE detected by coronary angiography ranges from 0.3% to 4.9% ^[2-10]^. In many studies, it has been reported that CAE is most frequently seen in RCA, followed by LAD, Cx and left main coronary artery (LMCA) involvement, respectively ^[11]^. In our study, CAE was detected most frequently in RCA, which is consistent with the literature. On the other hand, type 1 and type 3 ectasia were found more frequently in studies that examined the prevalence of individual CAE types ^[12]^. Type I ectasia was most commonly observed in our study. In addition, although it has been reported in some publications that CAE is more common in men, others have found no gender differences ^[12]^. In the present study, no statistically significant difference was found between the two genders in terms of the presence of CAE.

The association between CAE and inflammation has been frequently reported in the literature. Inflammation is the main pathophisiological mechanism underlying CAE. CAE is more common in human immunodeficiency virus (HIV) infection ^[13]^, and is associated with higher levels of uric acid, high-sensitivity C-reactive protein (hs-CRP) ^[14]^, and other inflammatory cytokines such as interleukin-1b, tumor necrosis factor alpha (TNF-α), and interleukin 10 ^[15]^. Monocyte to high-density lipoprotein ratio (MHR) ^[16]^, neutrophil to lymphocyte ratio (NLR) ^[17]^ and insulin-like growth factor I (IGF-I) level ^[18]^ are also elevated in CAE. These inflammatory processes may be independent of the presence of atherosclerosis. It was found that, in patients with CAE without atherosclerosis, hs-CRP levels are elevated compared to normal coronaries ^[19]^.

The mechanism for ischemia development is not clear in isolated CAE. Slow or turbulent flow in the enlarged vessel may cause thrombosis in the ectatic segment or embolism to the distal coronary artery ^[20]^. Güleç et al. ^[21]^ reported that, in cases of CAE, microvascular perfusion is also impaired in addition to epicardial perfusion. Two recent studies have suggested that left ventricular diastolic dysfunction was detected in patients with CAE examined by tissue Doppler imaging ^[22]^, which may be caused by microvascular dysfunction and/or ischemia ^[23]^.

There are many studies in the literature reporting that HT may play a role in the pathogenesis of CAE ^[24]^. Contrastingly, there are also studies reporting that the frequency of HT is not different from the control group ^[25]^. In our study, the HT prevalence could not be compared since there was no control group without CAE; however, 44.7% (n=64) of 143 patients with ectasia were diagnosed with HT.

CAE can present with symptoms caused by isolated or concomitant atherosclerosis. In the literature, CAE has been reported to cause slow flow, thrombus formation and vasospasm in the coronary arteries. It has been suggested that CAE causes clinical presentations that can lead to ischemic heart disease or even myocardial infarction without obstructive CAD. In our study, the presence of atherosclerosis was investigated in addition to CAE. Concomitant CAD was detected in 60.8% of the patients, and RCA was most commonly affected by stenosis and atherosclerotic lesions. Giannoglou et al. ^[26]^ reported CAD at a rate of 87.1% in patients with CAE.

MACE are well-established consequences of CAE ^[27]^. A three-year follow-up study showed similar clinical outcomes when compared to heavy atherosclerotic coronary burden ^[26]^. On the other hand, other studies reported non-benign courses in CAE as a result of dilated lumens with disrupted flow, a substrate for potential thrombus formation ^[27]^. In our study, patients with CAE who had significant coronary stenosis, a sign of severe atherosclerosis, showed a higher mortality rate in the long term, with similar trends in hospital admissions with chest pain, acute coronary syndrome and arrhythmia. Perhaps the long-term outcome of our patients provides stronger evidence for worse clinical outcome in the CCAE group compared to the ICAE group. In our study, the ejection fraction was lower in the group with recurrent interventions in patients with CAE. Concomitant significant coronary artery stenosis was more common and CV mortality was also higher in this group. Diabetes, HT and other factors were not predictors of recurrent intervention in the CAE group.

In our study, 12 of the 143 patients died after an average follow-up of 57 months, and the annual mortality rate found was 1.66%. There is limited data in the literature regarding the prognosis of patients with CAE. It is noteworthy that Markis et al. ^[8]^ reported in their study that the short-term prognosis was the same as that of patients with three-vessel disease followed up by a medical follow-up decision, and the annual mortality rate was reported at 15%. In another study, the mortality rate was not different from the control group, regardless of the form of treatment (medical or surgical) ^[5]^. Hartnell et al. found an annual mortality rate of 4.6% in patients followed by medical treatment and 2.4% in the surgical group, and concluded that the presence of CAE alone did not affect mortality.

It is known that CAE is one of the secondary causes of coronary slow flow. CSFP is most common in patients presenting with symptoms of chest pain, rest or mixed-pattern angina, and this is a hallmark of CSFP. Moreover, CSFP patients may present with life-threatening arrhythmias and sudden cardiac death ^[29,30]^. Although there are publications stating that male gender is a predictor for CSFP, data on the absence of a gender relationship are also available in the literature ^[31]^. However, in our study, there was no statistically significant difference between genders in terms of coronary CFSP. Little is known about the prognosis of CSFP, because the most published literature includes patients with known heart failure and other diseases and, as a result, its outcome is unclear ^[31]^. Sadamatsu et al. ^[32]^ and Chaudhry et al. ^[33]^ reported that patients with CSFP had a favorable long-term prognosis, while Fragasso et al. ^[34]^ investigated 12 patients with CSFP in an average follow-up of 15 years and concluded that patients with CSFP had a worse cardiac prognosis and, therefore, should be carefully followed. Moreover, the number of patients reported was limited (from a dozen to over a hundred); thus, CSFP remains poorly understood. Compared to previously published reports, our study had a relatively adequate sample size and explored risk factors for worse prognosis for the ﬁrst time in the literature.

Another important finding of our study is that hospitalization is more common in the presence of coronary slow flow in CAE. The presence of CAE may be one of the causes of CFSP, because one of the factors affecting the coronary artery blood flow is the geometric shape and the structure of the vein. It is known that CFSP can cause chest pain during rest and exercise and can be associated with increased frequency of hospitalization.

Limitations

Our study has some limitations. This is a single cohort study (without a control group) that required data collection for approximately 60 months. One of our limitations was the lack of treatment options for patients followed up with medical therapy. Further large prospective studies are needed to better demonstrate the criteria for treatment selection and prognostic evaluation in patients with CAE.

## CONCLUSION

The coronary slow flow phenomenon accompanying CAE may affect clinical follow-up, cause symptoms and may be a predictor of hospitalization. Significant atherosclerotic plaques coexisting with CAE may be predictive for MACE.

**Table:** 

Authors' roles & responsibilities	
MK	Substantial contributions to the conception or design of the work; or the acquisition, analysis or interpretation of data for the work; drafting the work or revising it critically for important intellectual content; final approval of the version to be published
ÖÖA	Drafting the work or revising it critically for important intellectual content; final approval of the version to be published
FY	Substantial contributions to the conception or design of the work; or the acquisition, analysis or interpretation of data for the work; drafting the work or revising it critically for important intellectual content; final approval of the version to be published
GIK	Drafting the work or revising it critically for important intellectual content; final approval of the version to be published
MT	Drafting the work or revising it critically for important intellectual content; final approval of the version to be published
